# O Desafio da Avaliação do Risco de Morte Súbita Cardíaca em Pacientes com Insuficiência Cardíaca de Etiologia Não Isquêmica

**DOI:** 10.36660/abc.20210633

**Published:** 2021-09-01

**Affiliations:** 1 Hospital Universitário Pedro Ernesto Rio de Janeiro RJ Brasil Hospital Universitário Pedro Ernesto, Rio de Janeiro, RJ – Brasil; 2 Hospital Unimed-Rio Rio de Janeiro RJ Brasil Hospital Unimed-Rio, Rio de Janeiro, RJ – Brasil

**Keywords:** Insuficiência Cardíaca/fisiopatologia, Morte Súbita, Disfunção Ventricular Esquerda, Galectina-3, Fibrose Endomiocárdica, Mortalidade, Biomarcadores

A Morte Súbita Cardíaca (MSC) é responsável por cerca de 50% das mortes de pacientes com cardiopatia isquêmica e não-isquêmica na presença de disfunção sistólica grave do ventrículo esquerdo.^1^ Diversos ensaios clínicos^2^^–^^8^ avaliaram a efetividade do Cardiodesfibrilador Implantável (CDI) na prevenção primária da MSC nos últimos 20 anos.

Os estudos MADIT II^2^ e SCD-HeFT^3^ estabeleceram as bases para a indicação^1^^,^^4^ do CDI na profilaxia primária da MSC dos paciente com insuficiência cardíaca (IC) de etiologia isquêmica. Por outro lado, ensaios clínicos que avaliaram pacientes com IC de etiologia não-isquêmica^3^^,^^5^^–^^8^ demonstraram resultados heterogêneos, e até o momento, lidamos com incertezas sobre quais os melhores candidatos para este tipo de terapia.

O SCD-HeFT^3^ avaliou o emprego do CDI para profilaxia de MSC em pacientes com IC de etiologia isquêmica e não isquêmica. Apesar dos benefícios do CDI observados no subgrupo de não-isquêmicos, os resultados foram inconsistentes. No estudo DEFINiTE^5^ o CDI não proporcionou redução de mortalidade significativa. Já os estudos AMIOVIRT^6^ e CAT^7^ apresentaram casuística reduzida e tempo curto de seguimento, produzindo resultados inconclusivos.

Uma metanálise^8^ desses quatros estudos, publicada em 2010, mostrou resultado favorável ao uso de CDIs neste grupo de pacientes, o que tem respaldado sua indicação nas diretrizes.^1^^,^^4^ No entanto, em 2016, o estudo DANISH^9^ reacendeu a discussão ao não observar redução de mortalidade em pacientes com IC não isquêmica submetidos a implante de CDI. Apesar das críticas sofridas,^10^ o estudo reforçou a necessidade da busca por preditores de melhor resposta ao implante de CDI.

Nesta edição dos Arquivos Brasileiros de Cardiologia, Kochi et al.^11^ avaliaram o papel da galectina-3 na predição de eventos arrítmicos graves e morte por todas as causas em pacientes com IC de etiologia não-isquêmica.^11^ Esta molécula vem sendo estudada como um marcador prognóstico em pacientes com cardiopatia e fibrose miocárdica.^4^^,^^12^^,^^13^ Sendo a fibrose um conhecido substrato para arritmias ventriculares, a quantificação do grau de acometimento ventricular por meio de biomarcadores que identificam tal alteração poderia facilitar a seleção de pacientes sob maior risco de MSC.

Apesar do racional e desenho elegantes do estudo, a galectina-3 não foi capaz de predizer MSC como variável contínua ou estratificada em quartis. Seu quartil mais elevado, no entanto, esteve associado a maior risco de morte por todas as causas.

A predição de risco de MSC nos pacientes com IC não isquêmica segue um desafio e novos estudos são necessários para identificar o paciente com maior probabilidade de se beneficiar do emprego do CDI.
