# Supplementary material for: O Desafio da Avaliação do Risco de Morte Súbita Cardíaca em Pacientes com Insuficiência Cardíaca de Etiologia Não Isquêmica
Source: Arq Bras Cardiol. 2021 Sep 1;117(3):542–3. [Article in Portuguese] doi: 10.36660/abc.20210633 (PMC8462945; doi:10.36660/abc.20210633)
Supplement: Translation

# Translation: The Challenge of Assessing Sudden Cardiac Death Risk in Patients with Nonischemic Heart Failure

**Affiliations:** 1 Rio de Janeiro RJ Brazil Hospital Universitário Pedro Ernesto, Rio de Janeiro, RJ – Brazil; 2 Rio de Janeiro RJ Brazil Hospital Unimed-Rio, Rio de Janeiro, RJ – Brazil

**Keywords:** Heart Failure/physiopathology, Death, Sudden, Cardiac, Ventricular Dysfunction, Left, Galectin 3, Endomyocardial Fibrosis, Mortality; Biomarkers

Sudden cardiac death (SCD) accounts for approximately 50% of deaths in patients with ischemic and nonischemic heart disease in the presence of severe left ventricular systolic dysfunction.^1^ Many clinical trials^2^^–^^8^ have evaluated the effectiveness of implantable cardioverter defibrillators (ICDs) in primary prevention of SCD in the last 20 years.

The Multicenter Automatic Defibrillator Implantation Trial II (MADIT II)^2^ and the Sudden Cardiac Death in Heart Failure (SCD-HeFT)^3^ defined the principles for indication^1^^,^^4^ of ICDs in primary prophylaxis of SCD of patients with heart failure (HF) of ischemic etiology. On the other hand, clinical trials evaluating patients with HF of nonischemic origin^3^^,^^5^^–^^8^ have shown heterogeneous results and, so far, there are uncertainties on the best candidates for this type of therapy.

The SCD-HeFT^3^ evaluated the use of ICDs in the prophylaxis of SCD in patients with HF of ischemic and nonischemic origin. Despite the benefits of ICDs observed in the nonischemic subgroup, the results were inconsistent. In the DEFINiTE,^5^ the use of ICD did not promote a significant reduction in mortality. The AMIOVIRT^6^ and the CAT^7^ had small sample sizes and short follow-up, yielding inconclusive results.

A meta-analysis^8^ of these four studies, published in 2010, described favorable results for the use of ICDs in this group of patients, which has supported the indication of these devices in the guidelines.^1^^,^^4^ However, in 2016, the DANISH^9^ study reopened the discussion as it did not demonstrate a reduction in mortality of nonischemic HF patients with the use of ICDs. Despite the critics received,^10^ the study reinforced the need for establishing the best predictors of response to ICD implantantion.

In this issue of the Arquivos Brasileiros de Cardiologia, Kochi et al.^11^ evaluated the role of galectin-3 in predicting severe arrhythmic events and all-cause mortality in patients with HF of nonischemic etiology.^11^ This molecule has been investigated as a prognostic marker in patients with HF and myocardial fibrosis.^4^^,^^12^^,^^13^ Fibrosis is a known substrate for ventricular arrhythmias, and the quantification of ventricular dysfunction using biomarkers that identify these changes would help the selection of patients at high risk of SCD.

Despite the elegant study rational and design, galectin-3 could not predict SCD neither as a continuous variable nor when stratified in quartiles. However, the highest quartile was associated with higher all-cause mortality.

The prediction of risk of SCD in nonischemic heart failure patients is still a challenge, and studies determining which patients would benefit most from the use of ICD are still needed.
